# Canadian vaccine safety surveillance reports following immunization with seasonal influenza vaccines, 2021–2022

**DOI:** 10.14745/ccdr.v50i12a02

**Published:** 2024-01-01

**Authors:** Elissa Giang, Yuhui Xu, Thivya Naganathan, Natalia Abraham, Marie-Thérèse Bawolak, Battouli Said Salim, Ashley Weeks, Amanda Shaw, Susanna Ogunnaike-Cooke

**Affiliations:** 1 Centre for Immunization Surveillance, Infectious Diseases and Vaccination Programs Branch, Public Health Agency of Canada, Ottawa, ON; 2 Marketed Health Products Directorate, Health Canada, Ottawa, ON

**Keywords:** influenza, vaccine adverse event, pharmacovigilance, vaccine safety, adverse event following immunization, AEFI

## Abstract

**Background:**

Seasonal influenza vaccines (SIV) authorized for use in Canada have all undergone rigorous regulatory assessments for safety and effectiveness. Serious adverse events following immunization (AEFI) can occur, though they are rare. Continuous safety surveillance of vaccines during the post-marketing phase is a critical component of vaccination programs. This enables the detection of rare, late onset, or unexpected adverse events. An updated safety summary following the introduction of any new vaccines and/or formulations to immunization programs is necessary for refining the risk-benefit profile of a specific vaccine and maintaining public confidence. Here we provide an updated safety summary for SIVs distributed during the 2021/2022 influenza season from AEFI reports submitted to the Canadian Adverse Event Following Immunization Surveillance System (CAEFISS) and the Canadian Vigilance Database (CVD).

**Methods:**

We searched CAEFISS and CVD for individuals who were vaccinated with a SIV between October 1, 2021, and March 31, 2022. Descriptive statistics were calculated, including median age of vaccinated individuals, vaccines co-administered with SIV, and the most frequently reported AEFIs. Crude AEFI reporting rates were calculated by severity of the AEFI report, and SIV-type using doses distributed data. Medical reviews were conducted for reports including death, serious events (or outcomes) after SIV were administered alone, and selected adverse events (i.e., anaphylaxis, Guillain-Barré syndrome, febrile seizures, oculo-respiratory syndrome). Disproportionality analysis was used to identify potential safety signals among SIV and AEFI pairs.

**Results:**

There were 448 AEFI reports, with most AEFI classified as non-serious events (84.2%). The majority of reports described vaccination in adults at least 65 years of age (38.6%). The most frequently reported AEFIs were vaccination site pain, urticaria, pyrexia and rash. Medical review of AEFI reports did not find any evidence that reported deaths were related to vaccination with SIV. Among serious reports, nervous system disorders were the most commonly reported medical conditions. A higher number of events related to vaccination errors were also identified using disproportionality analysis.

**Conclusion:**

Findings from our analysis of reports to CAEFISS and CVD following vaccination with SIV are consistent with the known safety profile of SIVs distributed during the 2021/2022 influenza season. The majority of reports were non-serious with the most common AEFI symptoms occurring at the vaccination site or systemic symptoms that were self-limiting. The majority of vaccination error reports involved the administration of the vaccine at an inappropriate site, although no serious AEFIs were reported.

## Introduction

Annual vaccination with seasonal influenza vaccine (SIV) remains the most effective strategy to decrease morbidity and mortality of influenza virus infection ((1)). In Canada, annual influenza vaccination is recommended for anyone six months of age and older, with a focus on targeted groups at highest risk of influenza-related complications ((1)). Several influenza vaccines are currently authorized for use in Canada; during the 2021/2022 influenza season, five types of SIVs were distributed in Canada, including standard dose (IIV4-SD) and high-dose (IIV4-HD) egg-based quadrivalent inactivated influenza vaccine, standard-dose cell culture-based quadrivalent inactivated influenza vaccine (IIV4-cc), quadrivalent live attenuated influenza vaccine (LAIV4), and adjuvanted egg-based inactivated influenza vaccine (IIV3-Adj) ((1)). Among these SIVs, the most frequently reported non-serious adverse events following immunization (AEFI)s in both children and adults were vaccination site reactions (e.g., pain, redness, swelling) and self-limiting systemic symptoms (e.g., fever, headache, nausea) ((1)).

All influenza vaccines authorized in Canada are considered to be safe ((1)); nevertheless, routine safety monitoring of AEFIs following SIV remains an essential component of annual influenza immunization programs. Notable AEFIs of concern include immediate hypersensitivity reactions such as anaphylaxis, Guillain-Barré syndrome (GBS), oculo-respiratory syndrome (ORS) and febrile seizures ((1)). Anaphylaxis reactions may be due to either an active component or additive in the vaccine; however, true anaphylaxis reactions are rare and occur at a rate of one per million doses for many vaccines ((2)). A proven association between use of the pandemic swine flu vaccine and GBS was identified in 1976 in the United States, which paused this particular vaccination campaign ((3)). Since 1976–1977, GBS has not been consistently associated with influenza vaccines; however, there is a need to monitor for GBS occurrence during mass vaccination campaigns, particularly following the use of pandemic influenza vaccines ((4)). Minor unexpected AEFIs have also been reported in Canada, including reports of ORS following receipt of past influenza vaccines ((5)). There is a small increased risk for febrile seizures when an inactivated influenza vaccine is administered during the same visit with PCV13 (pneumococcal) vaccine or the diphtheria, tetanus and pertussis (DTaP) vaccine ((6)). However, the risk of febrile seizures with any combination of these vaccines is small (up to 30 febrile seizures per 100,000 children vaccinated) and should be interpreted in the context of preventing pneumococcal and influenza infections among children ((6)).

Although rare, serious and unexpected AEFIs can occur following SIV. Post-marketing vaccine safety surveillance to identify late onset, rare or unexpected AEFIs is critical to any immunization program to enable effective public health action and to maintain vaccine confidence and public trust. The Public Health Agency of Canada (PHAC) and Health Canada share the monitoring of the quality, safety and effectiveness of vaccines marketed in Canada. At the national-level, AEFI reports are received in two safety surveillance systems: Canadian Adverse Event Following Immunization Surveillance System (CAEFISS) ((7)) (managed by PHAC); and Canada Vigilance Database (CVD) ((8)) (coordinated by Health Canada). The objective of this report is to summarize influenza AEFI reports received in both CAEFISS and CVD to assess the general safety profile of SIVs distributed during the 2021/2022 influenza season and to compare SIV safety trends over time.

## Methods

### Data sources

The CAEFISS is managed by PHAC and involves both passive and active surveillance monitoring systems that are designed to detect rare, late onset or unexpected events for any authorized vaccine in Canada ((7)). The CAEFISS receives spontaneous (passive) AEFI reports from federal, provincial and territorial public health authorities. Active surveillance is conducted through the Canadian Immunization Monitoring Program, ACTive (IMPACT) by nurse monitors under the supervision of paediatric and/or infectious disease medical specialists ((9)). The AEFI reports submitted to CAEFISS do not imply a causal relationship between the vaccine and AEFI, but that reported events are temporally associated with the vaccine (i.e., occur after vaccination within a biologically plausible timeframe) and have no other clear cause at the time of reporting. The AEFI form ((10)) submitted to CAEFISS collects information on sex, age, vaccines administered, doses and lot number, medical history and AEFIs experienced. AEFIs, including signs, symptoms and diagnoses, are assigned preferred terms (PT) by trained personnel using the Medical Dictionary for Regulatory Activities version current at the time of data collection ((11)). A systematic medical case review is conducted by trained health professionals who classify cases for reporting using standardized case definitions as applicable ((12)). A serious AEFI report is identified based on the International Council on Harmonisation of Technical Requirements for Registration of Pharmaceuticals for Human Use ((13)), as an event that results in death, is life-threatening, requires inpatient hospitalization or prolongation of existing hospitalization, results in persistent or significant disability/incapacity or results in congenital anomalies/birth defects. Any medical event that may not be immediately life-threatening but requires intervention to prevent one of the outcomes listed above may also be considered serious.

Health Canada coordinates CVD, which contains information about suspected adverse reactions to health products (including vaccines) that are submitted voluntarily by consumers and healthcare professionals. In addition, market authorization holders (manufacturers and distributors) are required to report serious AEFIs involving their marketed health products to CVD when they become aware of them. Specific reporting requirements for market authorization holders are described in the Food and Drug Regulations ((14)). The adverse reaction form collects information on reporter’s profession, age and sex of the vaccine recipient, vaccine(s) administered, the adverse reaction experienced and the level of care obtained. Signs and symptoms are coded using Medical Dictionary for Regulatory Activities ((11)) and reports are considered serious based on the definitions included in the Food and Drug Regulations ((14)) and the International Council on Harmonisation serious definition ((13)).

### Data analysis

We searched CAEFISS and CVD for AEFI reports among persons of any age who were vaccinated with SIV between October 1, 2021, and March 31, 2022 (analytic period). Our search included any report where SIV was administered alone or concomitantly with at least one other vaccine on the same day. Since healthcare providers and consumers can voluntarily submit AEFI reports to their federal, provincial and territorial public health authority (reports are sent to CAEFISS) and to CVD, there is the potential for duplicate reports between surveillance systems. To minimize duplicate reporting, initial groupings of CAEFISS and CVD reports were performed based on primary case information such as sex, age of the vaccinee, date the vaccine(s) was administered, vaccine trade name(s) and AEFIs to consolidate duplicate reports.

### Descriptive statistics

We described all reports submitted to CAEFISS and CVD for persons of any age who were vaccinated with SIV during the analytic period. Descriptive statistics were calculated, including median age and sex of the vaccine recipient, time-to-onset (the date from vaccination to onset of first symptoms), concomitant vaccine administrations with SIV, and the most frequently reported preferred terms. All data analyses were performed in R, the programming language for statistical computing (R version 4.1.3) ((15)).

Crude AEFI reporting rates were calculated for all reports and serious reports and by SIV-type (i.e., IIV3-Adj, IIV4-SD, IIV4-HD, IIV4-cc, LAIV-4) by dividing the number of AEFI reports received during the analytic period by the total number of SIV doses distributed in Canada during the 2021/2022 influenza season.

### Medical review of reports

All reports that described death following vaccination with SIV and all reports of serious AEFIs were medically reviewed (where medical records were available). For each report, we identified the primary AEFI that initiated the report and the respective System Organ Class (SOC) and preferred term(s) for the primary AEFI. In addition, we reviewed reports and accompanying medical information, where available, for selected AEFI conditions, which include reports of anaphylaxis, GBS, febrile seizures and ORS. Where applicable, Brighton Collaboration Case Definitions (BCCD) were applied to assess the level of case certainty ((16–18)).

### Disproportionality analysis

Using CAEFISS reports only, we calculated the information component (IC) statistic to identify AEFI and SIV pairings that were disproportionately reported during the 2021/2022 influenza season compared to the expected reporting in prior influenza seasons (2010/2011 through 2020/2021). Values greater than zero for the lower credibility interval endpoint of the IC (IC_0.25_) were considered statistically significant and were subject to medical review unless they were previously included in our list of selected AEFI conditions (see Methods, Medical review of reports). Significant values do not imply causality of an AEFI and vaccine pair but can suggest potential vaccine safety adverse events that require further medical investigation.

## Results

Our search identified 448 AEFI reports in CAEFISS and CVD following receipt of SIV during the surveillance period and are summarized in **Table 1**. Of these, 377 reports (84.2%) were considered non-serious AEFIs, while 71 (15.8.%), which included 13 reports of death, met the definition for serious case. The median age of vaccinees was 52 years (range: 5 months–104 years) and most reports described receipt of SIV in persons aged 65 years and older (38.8%), followed by children aged 5–17 years (21.9%). The median time-to-onset was one day (range: 0–15 days). The majority of reports were among females (65.2%) compared to males (34.4%). Of the 286 reports with SIV given alone, 31 (10.8%) were classified as serious.

**Table 1:** Characteristics of serious adverse events following immunization reports following seasonal influenza vaccine received in CAEFISS and CVD, October 1, 2021–March 31, 2022, N=448

Characteristic	N	%
**Surveillance system**		
CAEFISS	414	92.4
CVD	34	7.6
**Seriousness**		
Non-serious	377	84.2
Serious	71	15.8
**Sex**		
Female	292	65.2
Male	154	34.4
Not specified	2	0.4
Time-to-onset in days^a^, median (range)	1.0	0.0–15.0
Age (years), median (range)	52.0	0.5–104
**Age groups (years)**		
0–4	61	13.6
5–17	98	21.9
18–49	40	8.9
50–64	71	15.8
65 and older	173	38.6
Unknown/not specified	5	1.1
**Co-administrations**		
1 (seasonal influenza vaccine given alone)	286	63.8
2	116	25.9
3 or more	46	10.3
**Reporter profession**		
Consumer/non-health professional	28	6.3
Health professional (MOH/MHO, MD, RN, Pharmacist)	214	47.8
IMPACT	9	2.0
Unknown/Not specified	197	43.9

Abbreviations: CAEFISS, Canadian Adverse Event Following Immunization Surveillance System; CVD, Canadian Vigilance Database; IMPACT, Canadian Immunization Monitoring Program, ACTive; MD, doctor of medicine; MOH/MHO, medical officer of health; RN, registered nurse; SIV, seasonal influenza vaccine

^a^ Based on reports where time-to-onset was available

There were 162 reports (36.1%) that listed at least one additional vaccine administered on the same day as the SIV. The most commonly co-administered vaccines varied in accordance with age-based recommendations in the immunization schedule. Among children aged 0–4 years, the measles, mumps and rubella vaccine was the most frequently reported vaccine co-administered with SIV (60.5%), followed by diphtheria, tetanus, pertussis, polio and *Haemophilus influenzae* type b vaccine (DTaP-IPV-Hib, 36.8%) and meningococcal conjugate C vaccine (Men-C-C, 36.8%). Among adults aged 65 years and older, coronavirus disease 2019 (COVID-19) vaccines (67.1%) and pneumococcal polysaccharide 23 vaccines (Pneu-P-23, 31.6 %) were the most frequently co-administered with SIV. Reports describing co-administration are not mutually exclusive, as vaccine recipients can receive more than one co-administered vaccine on the same day. The 10 most frequently reported AEFIs are shown in **Table 2** and included vaccination site pain (17.4%), urticaria (11.6%), rash (10.7%) and pyrexia (10.7%).

**Table 2:** Most frequently reported Medical Dictionary for Regulatory Activities preferred terms following administration with seasonal influenza vaccines, by seriousness, October 1, 2021–March 31, 2022

MedDRA preferred term	N	%^a^
Vaccination site pain	78	17.4
Urticaria	52	11.6
Pyrexia	48	10.7
Rash	48	10.7
Vaccination site erythema	43	9.6
Vaccination site swelling	43	9.6
Pruritus	39	8.7
Erythema	37	8.3
Vomiting	36	8.0
Dyspnea	33	7.4

Abbreviation: MedDRA, Medical Dictionary for Regulatory Activities

^a^ Percentage calculated as a proportion of total reports for each MedDRA term

**Figure 1:**
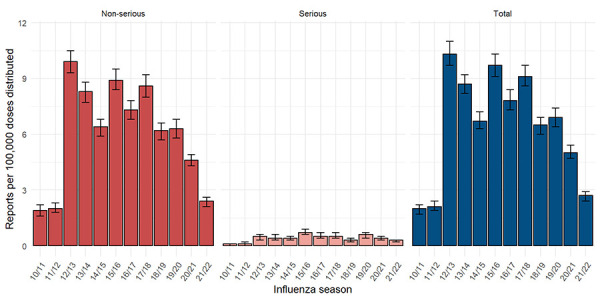
Crude adverse event following immunization reporting rate^a^ following receipt of seasonal influenza vaccine by seriousness, 2010/2011 to 2021/2022 influenza seasons^b^ a Per 100,000 doses distributed b Canadian Adverse Event Following Immunization Surveillance System reports only

During the 2021/2022 influenza season, 15,605,176 SIV doses were distributed in Canada. Based on only the 414 CAEFISS reports per Table 1 (as historical information from CVD was not available), the overall crude reporting rate in 2021/2022 was 2.7 reports per 100,000 doses distributed. Similarly for serious reports, with 41 serious reports from CAEFISS, the crude reporting rate was 0.3 per 100,000 doses distributed. Since the 2019/2020 influenza season, there has been a consistent downward trend in reporting rates among total and serious reports, with non-overlapping confidence intervals (**Figure 1**).

Based on SIV-type, crude reporting rates were highest among IIV4-HD vaccine (4.2 reports per 100,000 doses distributed), followed by IIV3-SD vaccines (2.2 reports per 100,000 doses distributed), while among other SIV-types, fewer than 10 reports were reported (**Table 3**). Across all SIV-types, the reporting rate for serious events remained low, with one or fewer report per 100,000 doses distributed.

**Table 3:** Description of adverse event reports following receipt of seasonal influenza vaccine-type, N=389^a^

Report descriptor	Influenza vaccine type				
	IIV3-Adj	IIV4-SD	IIV4-HD	IIV4-cc	LAIV-4
Total reports	Fewer than 10	296	79	Fewer than 10	Fewer than 10
Non-serious	SDC	262	67	SDC	SDC
Serious	SDC	34	12	SDC	SDC
Proportion serious	SDC	11.5%	15.2%	SDC	SDC
Total doses distributed	69,587	13,456,161	1,876,475	34,533	168,420
Reporting rate (95% CI)^b^	SDC	2.2 (2.0–2.5)	4.2 (3.3–5.2)	SDC	SDC
Serious rate (95% CI)	SDC	0.3 (0.2–0.4)	0.6 (0.3–1.1)	SDC	SDC

Abbreviations: CI, confidence interval; IIV3-Adj, adjuvanted egg-based trivalent inactivated influenza vaccine; IIV4-cc, standard-dose cell culture-based quadrivalent inactivated influenza vaccine; IIV4-HD, high-dose egg-based quadrivalent inactivated influenza vaccine; IIV4-SD, standard-dose egg based quadrivalent inactivated influenza vaccine; LAIV4, quadrivalent live attenuated influenza vaccine; SDC, statistical disclosure control (suppression of small cell counts)

^a^ Fifty-seven reports with missing influenza vaccine name and type, and two reports with vaccines distributed in previous season were excluded

^b^ Rates were calculated per 100,000 doses distributed

### Deaths

There were 13 reports of death following vaccination with SIV. Medical information (e.g., patient medical history, clinical information leading up to events, pre-existing and concurrent comorbidities) was available for 10 of these reports. Of these, neurocognitive disorders were the most cited cause of death (n=4/10, 40%). Six reports did not provide cause of death or results of further investigations (at the time of reporting), but all six reports described pre-existing medical conditions including chronic obstructive pulmonary disease, coronary artery disease, diabetes, hypertension and a genetic disorder. The majority of deaths (n=9/13, 69%) occurred among adults aged 65 years and older, and the median age was 84 years (range: 12 months–98 years). The remaining three reports had insufficient information on cause of death, patient medical history or clinical information leading up to events. Of those with sufficient information for causality assessment, none were deemed to be consistent with a causal association with vaccination.

### Serious reports

There were 31 reports classified as serious following vaccination with SIV only. The most common SOC was nervous system disorders, including diagnosis of GBS (two reports), seizure (one report), syncope (one report) and petit mal epilepsy (one report). There was one AEFI report identified for the following SOCs: gastrointestinal disorders (one report of abdominal pain); general disorders and administration site conditions (one report of chest pain); immune system disorder (one report of event managed anaphylaxis); investigations (one cerebrospinal fluid test abnormal); respiratory, thoracic and mediastinal disorders (one report of dyspnea); and skin and subcutaneous tissue disorders (one report of urticaria). The remaining 20 reports did not identify the primary event that prompted the report; therefore, the respective SOC and preferred term for the primary event could not be determined.

### Selected adverse events

Our search did not find any reported cases of ORS following receipt of a SIV distributed during the 2021/2022 influenza season. Twenty-nine possible reports of select AEFIs (i.e., anaphylaxis, GBS, febrile seizures) were identified from our search and are further described below.

**Anaphylaxis:** There were 16 reports that had at least one PT suggestive of anaphylaxis, most of which (n=12/16, 75%) described anaphylaxis following receipt of SIV alone. In 14 of the 16 (87.5%) reports, IIV4-SD was the SIV-type received. Only six reports met the BCCD criteria. One report was classified as level 1 (highest level of diagnostic certainty) and five reports were classified as level 2. The remaining 10 reports did not contain sufficient information to either assign a certainty between BCCD levels 1–3 or rule out anaphylaxis (i.e., BCCD level 5). Further, there was no information within these reports to confirm whether the reactions experienced (at time of reporting) were immunoglobulin E (IgE)-mediated. Among the six confirmed reports, the median time-to-onset was 43 minutes (range: 4–230 minutes) and most reports (n=12/16, 75%) described anaphylaxis following receipt of SIV alone.

**Guillain-Barré syndrome:** Four reports of possible GBS were identified following receipt of a SIV. After applying the BCCD, one report was classified as level 3; the remaining three reports could not be classified according to BCCD, mainly due to incomplete medical information within the AEFI report. The time-to-onset was nine days, and the single report described GBS after receipt of more than one vaccine.

**Febrile seizures:** There were nine possible reports suggestive of a febrile seizure. Four reports were considered serious: following medical review, all four reports were classified as BCCD level 1 and all four reports occurred among children two years of age and younger and a median age of one year (range: 1–1.5 years). Among these reports, the time to onset for all reports was one day. All four reports listed SIV concomitantly administered with more than one childhood vaccine, including measles, mumps and rubella, pneumococcal conjugate 13 (Pneu-C-13), varicella, DTaP-IPV-Hib and Hepatitis B (HB) vaccines.

### Disproportionality analysis

When compared to previous influenza seasons, two preferred terms were reported more frequently this season than expected in the CAEFISS database: “vaccination error” and “lymphadenopathy”. There were 15 reports of vaccine error, where the frequency of observed reports was 2.7 times (IC/IC_0.25_: 1.45/0.57) the proportion of reports received in prior influenza seasons in CAEFISS. The most common reason for vaccine error was vaccine administration at an inappropriate site (n=13/15, 86.7%), while the remaining report indicated the vaccine was given outside the recommended age indication. Further review of vaccine error reports identified AEFI conditions consistent with shoulder injury related to vaccine administration (SIRVA; i.e., joint range of motion decreased, shoulder injury, joint movement impairment); however, all reports were considered non-serious.

There were 13 reports of lymphadenopathy, where the frequency of observed reports was 2.1 times higher (IC/IC_0.25_: 1.10/0.16) than the proportion of lymphadenopathy reports in CAEFISS observed in prior influenza seasons. Given that the majority of AEFIs reports were non-serious, and lymphadenopathy has been reported as an AEFI during clinical trials ((19)) and during post-approval use among SIV ((20)), no further medical review was conducted.

## Discussion

This article describes reports to CAEFISS and CVD following receipt of SIV between October 2021 and March 2022. Most reports were for adults aged 65 years and older (38.6%), the group for which SIV is routinely recommended due to increased risks for complications from influenza, including hospitalizations and death ((1)). Overall, AEFIs reported were consistent with the known safety profile of SIV, characterized by pre-licensure studies and post-marketing surveillance, and included vaccination site reactions (i.e., pain, swelling, erythema) and systemic reactions (i.e., fever, nausea, vomiting). The overall and non-serious reporting rates were significantly lower than those observed during previous influenza seasons. Given that the annual SIV campaign is not new, vaccine recipients and providers may be less likely to report milder and less serious AEFIs with vaccines they are familiar with, an epidemiological phenomenon referred to as the Weber effect ((21)). There is some evidence of an observed reduction in non-serious spontaneous reporting due to the COVID-19 pandemic, which may be driven primarily by changes in reporting practices by healthcare professionals or vaccine recipients. Our data supports this observation as the reduction is mainly seen in non-serious reports, while serious reporting rates remain within historic ranges ((22)). The data are also comparable to other studies looking into the safety of SIVs in other countries ((1,23–25)).

Based on the medical information available, most reports of death following receipt of SIV were due to neurocognitive disorders. There were six reports (out of the 13 reports with an outcome of death) that indicated a broad range of pre-existing medical conditions that may have contributed to increased risk of severe clinical outcomes of influenza infection, including death. Among these reports, we did not identify any patterns or further evidence to suggest a causal relationship between vaccination and death. Because reports received in CAEFISS and CVD do not always contain complete medical information on the patient, we were unable to assess for potential confounders (i.e., pre-existing medical conditions, medications) or for causality for some of these reports.

Among reports classified as serious, the most commonly reported AEFIs following vaccination of SIV alone were classified as nervous system disorders and are mentioned in the associated SIV product monographs as having been reported after vaccination but for which causal association is unknown ((19,26)). Reassuringly, their occurrence remained rare, though providers are expected to communicate risk-benefit information to vaccine recipients and caregivers along with advice for what to do if such an event occurs ((1)).

There was disproportionate reporting of vaccine error terms, with the majority of reports describing symptoms consistent with SIRVA. Shoulder injury related to vaccine administration typically occurs moments to days after the vaccine is injected and can result in prolonged and even permanent shoulder dysfunction. While the incidence of SIRVA is not well known, it is assumed to be uncommon ((27)).

## Limitations

It is important to consider the limitations of CAEFISS and CVD that are inherent to passive surveillance systems. These can include under-reporting, reporting bias, varied report quality and completeness, and appropriate denominator data for contextualizing the number of AEFI reports received. Currently, there is no mechanism for tracking the number of SIV doses administered nationally. In the absence of such data, doses of SIV distributed served as a proxy for doses of SIV administered; therefore, crude reporting rates should be interpreted with caution. Further, absence of an unvaccinated control group makes it difficult to examine the association between an AEFI and the vaccine(s). It is rarely possible to determine causality based on reports submitted to national passive surveillance systems alone. When a signal is detected, further investigation is always warranted at the individual and population level to determine causality ((28)). Nonetheless, these surveillance systems provide a means for identifying statistical safety signals for rare and unexpected AEFIs and can be used to make general conclusions of the safety of vaccines administered in Canada.

## Conclusion

The overall goal of post-marketing safety surveillance is to detect rare or unusual safety concerns that may signal previously unknown associations between a given vaccine and AEFIs, or changes in expected safety profiles in terms of frequency or severity of selected AEFIs. Based on this updated summary, we did not observe any trends or patterns of concern following receipt of a SIV in Canada during the 2021/2022 influenza season. Our findings are consistent with data from pre-licensure clinical trials and post-licensure safety assessments and support that SIV exhibit a favourable safety profile ((1,23,29)).
